# Biomineralization of Human Genomic DNA into ZIF-8, a Zeolite-Like Metal-Organic Framework

**DOI:** 10.17691/stm2024.16.1.01

**Published:** 2024-02-28

**Authors:** I.H. Shaykhutdinov, P.V. Iliasov, L.V. Limareva, A.S. Sustretov, D.A. Kokorev, A.V. Sokolov

**Affiliations:** Researcher, Laboratory of New Medical Materials and Technologies, Professional Center for Education and Research in Genetic and Laboratory Technologies; Assistant, Chemistry Department, Pharmacy Institute; Samara State Medical University, 89 Chapaevskaya St., Samara, 443099, Russia; Leading Researcher, Laboratory of Non-Infectious Immunology, Professional Center for Education and Research in Genetic and Laboratory Technologies; Samara State Medical University, 89 Chapaevskaya St., Samara, 443099, Russia; Associate Professor, Head of the Laboratory of Non-Infectious Immunology, Professional Center for Education and Research in Genetic and Laboratory Technologies; Samara State Medical University, 89 Chapaevskaya St., Samara, 443099, Russia; Head of the Laboratory of Human Metagenomics, Professional Center for Education and Research in Genetic and Laboratory Technologies; Samara State Medical University, 89 Chapaevskaya St., Samara, 443099, Russia; Specialist, Laboratory of Human Metagenomics, Professional Center for Education and Research in Genetic and Laboratory Technologies; Samara State Medical University, 89 Chapaevskaya St., Samara, 443099, Russia; Head of the Laboratory of New Medical Materials and Technologies, Professional Center for Education and Research in Genetic and Laboratory Technologies; Samara State Medical University, 89 Chapaevskaya St., Samara, 443099, Russia

**Keywords:** biomineralization, encapsulation, metal-organic framework, human genomic DNA, genetic vectors, ZIF-8

## Abstract

**The aim of the study** was to assess the capabilities of human genomic DNA biomineralization into ZIF-8 metal-organic framework (MOF) preserving DNA sequence integrity after the encapsulation cycle and composite dissolving. The study is an initial stage of the project aimed at developing an abiotic vector to be used when working with native nucleic acids of an arbitrary size based on DNA@ZIF-8 composite.

**Materials and Methods:**

We studied human genomic DNA isolated from lymphocytes of peripheral blood of healthy volunteers using Proba-NK kit (DNA-Technology LLC, Russia). Genomic DNA purity and concentration was estimated spectrophotometrically at 260/280 nm using Tecan Infinity 200 Pro plate reader (Tecan Instruments, Austria). ZIF-8 was synthesized in the physiological conditions (37°C) by mixing zinc salt and 2-methylimidazole aqueous solutions at different molar ratios. Human genomic DNA was encapsulated into ZIF-8 in similar conditions. The obtained MOF and DNA@ZIF-8 composite were studied using X-ray powder diffraction at the Phaser D2 XRPD device (Bruker, USA), and the specific surface area was estimated using Autosorb iQ porosimetry analyzer (Quantachrome, USA). The encapsulated DNA was quantified by dissolving DNA@ZIF-8 composite in the citrate buffer. DNA integrity was assessed by real-time allele-specific PCR (AS-PCR) using the kits for single nucleotide polymorphisms (Lytech, Russia) at the Quantstudio 6 Pro PCR machine (Thermo Scientific, USA). In case of using the kits with electrophoretic detection, the amplification was performed on the thermal cycler T100 (Thermo Scientific, USA).

**Results:**

The polymer ZIF-8 and DNA@ZIF-8 composite were obtained at different molar ratios of zinc ions and 2-methylimidazole. We characterized their structure and specific surface area. Genomic DNA biomineralization efficacy was found to be about 7–8%. PCR indicated the integrity of non-selectively chosen loci within the biomineralized DNA.

**Conclusion:**

The study confirmed the possibility of human genomic DNA encapsulation into ZIF-8 metal-organic framework. After the biomineralization, DNA was found to preserve feasibility to be used in studies to investigate genetic constructs.

## Introduction

One of the most important applied fields in gene engineering and therapy is the development of genetic material delivery systems [1]. Both biotic and abiotic vectors are used as delivery systems. Biotic vector systems, among which there are viral and plasmid vectors, are applied most frequently [2–8]. They easily penetrate through biological barriers, and are able to effectively transfect and induce long-term gene expression. However, viral vectors exhibit some major disadvantages, such as limited working load, the risk of carcinogenesis, immunogenicity, toxicity [9].

Among abiotic vector systems there are polymer [10], lipid [11, 12], magnetic [13], and gold-based nanoparticles [12]. Such delivery systems have a number of distinct advantages, in particular, an ability to load large-sized components, ease of formation, low toxicity, minimal immune response [14–16]. Moreover, production technologies of non-viral vector systems are easily scaled [17]. However, abiotic vectors are characterized to have some drawbacks restricting their implementation. So, many biodegradable polymers are known for low stability *in vivo*: e.g., liposomes are prone to spontaneous aggregation requiring the introduction of stabilizing agents into their structure [18]. In general, encapsulation of high-molecular bioorganic polymers (proteins, nucleic acids) using polymer and micellar carriers is related to practical difficulties due to low load efficiency [19], as well as major difficulties in overcoming extra- and intracellular membrane, and preserving functional integrity of encapsulated bioorganic compounds [20].

Recently, there have been the publications devoted to the use of metal-organic framework (MOF) as abiogenic genetic vectors. Mammalian cells were demonstrated [21] to be able to expose these polymers to endocytosis and ease the introduction of encapsulated or mineralized useful load in target cells, and do away with the need of using specialized transformation procedures. MOF are chemically and thermally stable, they can be formed under biocompatible conditions. One more factor to ease targeted delivery of useful load is MOF capability to protect genetic material against degradation in physiological conditions, and provide its controlled release [22]. So, zeolite-like imidazolate zincbased MOF ZIF-8 widely used for of biocomponents showed its biomineralization efficiency for proteins [23, 24], carbohydrates [25–28], viruses [29], and cells [30, 31], as well as plasmid DNA, microRNA [32, 33], nucleoproteins, and the components of genome editing systems [34–37]. However, there are no studies describing (genomic) DNA included in ZIF-8 and its analogues so far. It is worth noting that except high load bearing capacity, ZIF-8 is capable of breaking down in acid and weak-acid environmental pH [38, 39]. This characteristic of zinc imidazolate makes it promising for targeted drug delivery [40–45]. Some researchers have described the modifications of MOF-based composites by specific components, which ease its binding with targeted cell receptors, it additionally strengthening the potential of these carriers as the means of targeted drug delivery [35, 46].

**The aim of the study** was to assess the biomineralization efficiency of human genomic DNA into ZIF-8 MOF model in order to study ZIF-8 capabilities to work with native nucleic acids of arbitrary size.

## Materials and Methods

### Chemicals

We used 2-methylimidazole, puriss (Merck, Germany); zinc acetate tetrahydrate, extra pure (Vekton, Russia); zinc nitrate hexahydrate, extra pure (Vekton, Russia); deionized water (resistivity — 17.8 MOm·cm); propidium iodide (neoFroxx, Germany); citric acid, extra pure (Vekton, Russia), sodium citric acid tri-substituted 5.5-hydrate (Vekton, Russia), high-purity agarose (Acros Organics, USA), ethidium bromide (CDH, India).

### Obtaining ZIF-8

Having studied technical approaches enabling to synthesize ZIF-8 of high crystallinity in physiological conditions [47], we chose several techniques performed in aqueous solutions at 37°C, characterized by using zinc salt anion (i.e. those using zinc acetate or nitrate) and different mole ratio metal:ligand. Within the framework of the preliminary work, we tested the method with the reagent ratio of zinc acetate:2-methylimidazole:water equaled 1:1:≥50, and described in the work [48]. Despite of high MOF yield (over 70%), the obtained product was the mixture of crystalline phases, which was unacceptable with regard to the present study objectives. Therefore, in the experiments to study the characteristic of the polymer and its composite, we used three other mole ratios of the initial components of zinc nitrate: 2-methylimidazole:water, namely 1:15≥2000; 1:40:≥2000, and 1:60:≥2000 [49]. All experiments had ten replications.

### Native DNA obtaining and cleaning

Native genomic DNA was isolated from human peripheral blood lymphocytes using Proba-NK kit (DNA-Technology LLC, Russia) according to the manufacturer’s instruction. The obtained DNA was kept at –20°C. Before inclusion in MOF, we determined DNA purity and concentration by measuring optic density at 260/280 nm using Tecan Infinity 200 Pro plate reader (Tecan Instruments, Austria) with NanoQuant plate (Tecan Instruments, Austria).

### Obtaining genomic DNA@ZIF-8 composite

Genomic DNA, 800 μl, its concentration being 400 ng/μl, was added to zinc nitrate solution hexahydrate (concentration of 19.8 mg/ml) followed by adding 2-methylimidazole solution at concentration of 82 mg/ml, volume: 1.36 and 3.64 ml, 1 and 4 ml, which enabled to obtain the following mole ratios of MOF components — 1:40 and 1:60, respectively. The obtained suspension was shaken using a vortex-mixer for a minute and then incubated for 24 h at 37°C. After incubation, the samples were centrifuged for 10 min at 2000 g, the supernatant was being separated from precipitate; the precipitate was washed by deionized water (5 ml) four times and dried at 37°C.

### X-ray powder diffraction

The samples were studied using X-ray powder diffraction Phaser D2 with 1D detector LYNXEYE XE-T (Bruker, USA). The range of reflection angles 2θ was 2–35°.

### Measuring surface area by Brunauer– Emmett–Teller method (BET)

The measurements were performed using porosimeter Autosorb iQ (Quantachrome, USA), relative pressure being p/p0 9·10^–3^–0.995, at –196°C. Before the analysis, the samples were preliminary degasified in helium atmosphere at 60°C within 24 h.

### Quantitative assessment of genomic DNA after biomineralization

Propidium iodide solution was added to 96-well black flat bottom plate (SPL Life Sciences, Republic of Korea) containing DNA samples under study at the rate of 50 ng/well, and the total solution volume: 200 μl/well, incubated for 30 min at room temperature, and read fluorescence intensity at excitation wavelength of 535 nm, radiation wavelength being 617 nm on a plate reader Tecan Infinity 200 Pro (Tecan Instruments, Austria). The calibration function was recorded in DNA concentration range 0–100 ng/μl adjusted according to the measurements of DNA solution optic dentistry, at wavelength 260 nm on Tecan Infinity 200 Pro plate reader using NanoQuant plate (Tecan Instruments, Austria). The obtained ratios of fluorescence intensity dependency on DNA concentration were approximated by a linear function *y*=*a***·***x*+*b*, where *a* and *b* are constants, *y* is fluorescence intensity, *x* is DNA concentration, and calculated DNA concentrations in the samples according to the formula *x*=(*y*–*b*)/*a*. The calculations were made using Microsoft Office Excel 2016.

### PCR procedure

PCR was performed to assess DNA preservation after biomineralization in MOF ZIF-8. The citrate buffer (0.1 М; pH 5.0) was used to completely dissolve the composite; the buffer providing complete composite dissolving and DNA integrity preservation. After composite dissolving, DNA was purified by sorption and washing on magnetic particles using NK-Extra kit (TestGene, Russia).

Allele-specific PCR (AS-PCR) was performed using two methods: a real-time mode on a thermal cycler Quantstudio 6 Pro (Thermo Scientific, USA), and using electrophoretic detection. Single nucleotide polymorphisms (SNP) were chosen in the following genes: *AGT* (rs4762), *APOC3* (rs5128), *APOE* (rs429358), *IL1β* (rs1143627), *IL6* (rs1800795), *LIPC* (rs2070895), *LPL* (rs328), *MMP9* (rs11697325), *PON1* (rs662), *TNF-α* (rs1800629). PCR carrying conditions met the manufacturer`s instructions (Lytech Co. Ltd, Russia). Electrophoresis was performed in 3% agarose gel with ethidium bromide in accordance with the manufacturer’s recommendations (Lytech Co. Ltd, Russia), DNA fragments were visualized using gel-imaging system Vzglyad (Helikon, Russia). SNP data were on chromosomes 1, 2, 6, 7, 8, 11, 15, 19, 20 that enabled to assess nonspecific preservation of DNA integrity within a genome. Native DNA was used as control.

### Statistical data processing

The data were statistically processed using Microsoft Office Excel 2016 with AtteStat 11.5. The data adequacy for normal distribution was checked using a modified Kolmogorov criterion. Numeric data were presented as “mean value ± standard deviation”. To assess specific surface area, the correlation coefficient of calculated values was calculated automatically by the embedded software of Quantachrome ASiQwin 5.21 analyzer. The differences were considered statistically significant if p<0.05.

## Results and Discussion

### ZIF-8 investigation

**Figure 1.**
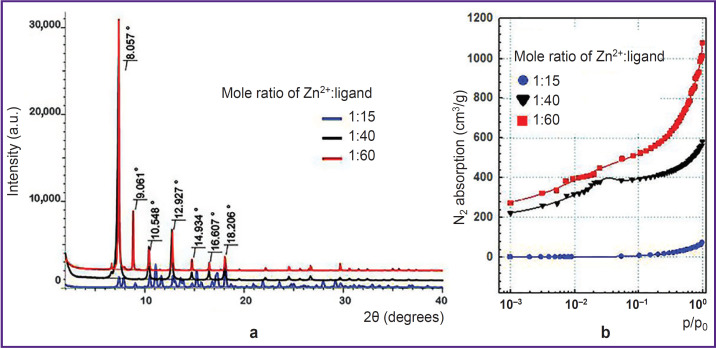
The study of ZIF-8 samples obtained in different mole ratios of zinc nitrate: 2-methylimidazole: (a) standard diffractograms; (b) standard isotherms of N_2_ absorption

During the synthesis, the samples were analyzed using X-ray powder diffraction (Figure 1 (a)). The obtained difractograms showed that samples with mole ratio Zn^2+^:ligand=1:40 and 1:60 have the characteristic reflection peaks at angles 2θ equal 8.0, 9.0, 10.5, 12.9, 14.9, 16.6, 18.2°; they are single-phase ZIF-8 samples with *sod*-topology and the slight share of amorphous phase. Moreover, the diffractogram of the sample with components ratio of 1:15 had numerous additional reflections testifying that the sample was the phase mixture.

The next stage included the polymer specific surface area measurement by BET method. Figure 1 (b) demonstrates the sorption N2 isotherms. The study showed the specific surface area for the samples ZIF-8 with mole ratio of components 1:15; 1:40, and 1:60 to be 7.0; 1570±132; 1854±173 m^2^/g, respectively. The correlation coefficient of the calculated values when using different p/p0 values was in the range of 0.984–0.999, the difference being significant if p<0.05. It should be noted that ZIF-8 used for microRNA encapsulation in the study [50] had similar surface area (1301.4 m^2^/g). The authors of the mentioned article thought the indicator to be rather high, and able to provide efficient effective inclusion of much useful load (up to 36 μg/mg of the polymer). However, for encapsulation of low-molecular compound, e.g. doxycycline, it would be enough to have specific surface area 75 m^2^/g [51].

Thus, the experiment demonstrated the sample with the component mole ratio of 1:15 to be characterized by low specific surface area. DNA mineralization under such conditions is inefficient. Moreover, the specific surface area of the other two samples were comparable and sufficient to carry out the experiments on DNA encapsulation, therefore, at next stage native DNA was exposed to mineralization at mole ratios of initial MOF components equal to 1:40 and 1:60.

### Analysis of DNA@ZIF-8 composite

**Figure 2.**
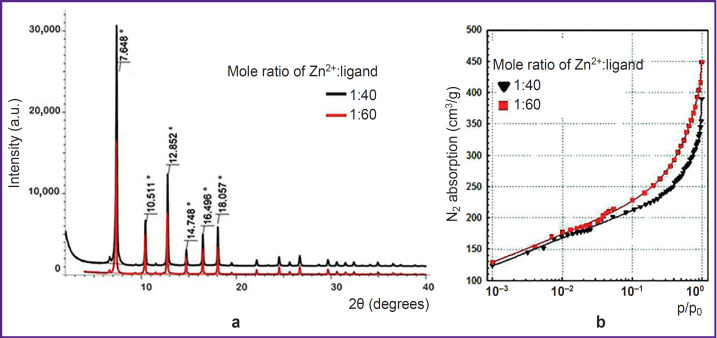
The study of DNA@ZIF-8 composite samples obtained in different mole ratios of zinc nitrate:2-methylimidazole: (a) standard diffractograms; (b) standard isotherms of N_2_ absorption

DNA@ ZIF-8 composites obtained according to the given description were studied using X-ray powder difraction (Figure 2 (a)). Difractometry data showed synthesized composites to have characteristic reflection peaks at the angles equal to 7.6, 10.5, 12.8, 14.7, 16.5, 18.0°; they were single-phase ZIF-8 samples consistent with literature data [48, 49].

The specific surface area of composites was studied under the similar conditions compared to those of ZIF-8 without DNA. Figure 2 (b) represents the sorption isotherms. The research findings showed the specific surface area for DNA@ZIF-8 composite samples with mole ratio of components 1:40 and 1:60 to be 524±54 and 715±74 m^2^/g, respectively. The correlation coefficient of the calculated values when using different p/p0 values was 0.999, the difference being significant if p<0.05.

Thus, DNA@ZIF-8 composite was found to have significant specific surface area decrease compared to pure ZIF-8 (see the Table). This fact can be explained by including genomic DNA into MOF structure and blocking the gas access to pores.

**Table:** Specific surface area of ZIF-8 and DNA@ZIF-8 composite

Mole ratios	ZIF-8 surface area (m^2^/g)	DNA@ZIF-8 surface area (m^2^/g)
1:40	1570±132	523±54
1:60	1854±173	715±74

Subsequently, dry DNA@ZIF-8 composite was dissolved in the citrate buffer (0.1 М; pH 5.0) in the volume of 1.6 ml and incubated for 24 h at 37°C. After dissolving we remeasured DNA concentration using propidium iodide solution. The use of DNA measurement using propidium iodide in this case was due to the fact that ligand of MOF — 2-methylimidazole, which is in excess in regard to DNA, is characterized by partial spectrum sorption in the range of 240–280 nm. The sorption made it impossible to use 260/280 nm spectrophotometry for quantitative DNA assessment in the samples in the present experiment [52].

The efficiency of DNA inclusion into MOF was calculated as follows:

X=C2⋅V2C1⋅V1⋅100%,

where *C*1 is DNA concentration before inclusion into MOF; *V*1 is DNA volume before inclusion into MOF; *C*2 is DNA concentration after the composite dissolving; *V*2 is the volume of composite solution in the citrate buffer.

The inclusion efficiency of genomic DNA into ZIF-8, for the samples with initial components ratio 1:40 and 1:60 was 8.58±1.50 and 7.94±1.25%, respectively. Moreover, there were found no significant differences in inclusion efficiency depending on mole ratio of MOF components (p>0.05). It should be noted that other authors in their studies [20, 50, 53, 54] appeared to achieve next higher order values of mineralization efficiency of plasmid DNA (46–82%) and microRNA (61–72%), and the work [51] demonstrated even higher (up to 90%) uploading efficiency of DNA/RNA complexes, which was achieved due to the fact that nucleic acids were exposed to electrostatic binding to surface articles of iron-containing MOF composite and doxycycline, rather than encapsulation. It is expected that comparatively low degree of DNA biomineralization in the present study was due to concurrent processes occurring simultaneously with DNA biomineralization itself, and perturbing its progress. Such processes include, firstly, the process of ZIF-8 independent polymerization, and, secondly, ZIF-8 nucleation processes around low-molecular compounds, which the samples under study had, with their following encapsulation. Furthermore, the molecules of plasmid DNA or microRNA are characterized by multiply smaller sizes and mass compared to a human full-scale chromosomal DNA that is likely to ease their building into MOF structures.

**Figure 3.**
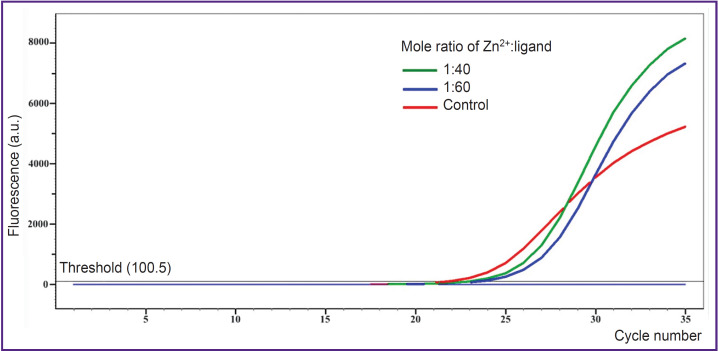
Amplification diagram of SNP rs4762 after biomineralization and dissolving DNA@ ZIF-8 composites in a citrate buffer Ct threshold cycle equals 21.5 (control), 23.1 (mole ratio 1:40), 23.8 (mole ratio 1:60)

**Figure 4.**
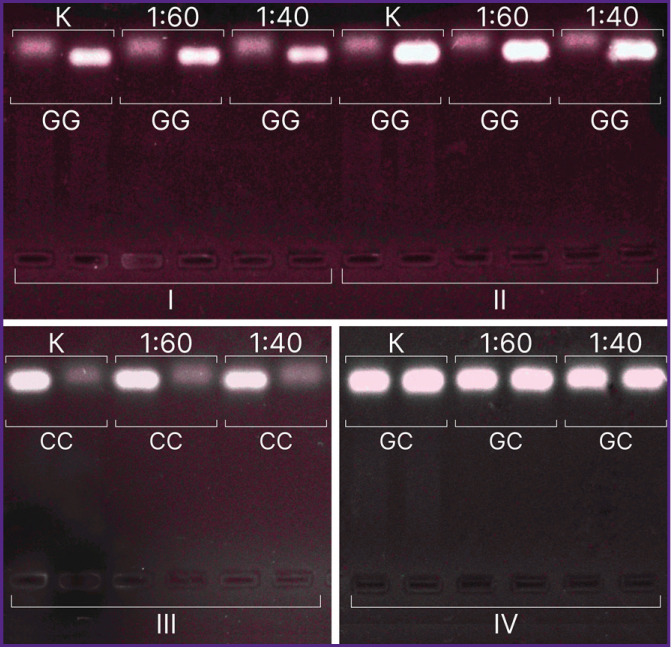
Electropherogram of PCR products in 1.5% agarose gel: C — control; 1:40, 1:60 — mole ratio of Zn^2+^: ligand; I — *MMP9* gene (8202 G>A, rs11697325); II — *TNF-α* gene (308 G>A, rs1800629); III — *IL1β* gene (31 T>C, rs1143627); IV — *IL6* gene (174 G>C, rs1800795)

According to AS-PCR in real-time mode for native DNA, we got the amplification curves corresponding to the presence of CC genotype for rs4762 polymorphism (Figure 3), CG — for rs5128, TC — for rs429358, AG — for rs2070895, CC — for rs328, GG — for rs662, and in case of electrophoretic detection — the findings were the following: genotype GG — for rs1800629, GG — for s11697325, CC — for rs1143627, GC — for rs1800795 (Figure 4). After DNA@ZIF-8 composites dissolving (the mole ratio of the components is 1:40 and 1:60) and performing PCR, the results were consistent with the genotype of native DNA in all the samples under study. AS-PCR findings confirmed the presence of randomly chosen DNA areas within the genome boundaries in the samples after biomineralization into ZIF-8. It indirectly indicates the significant part of genomic DNA was preserved during the encapsulation MOF destruction, as well as the capability of ZIF-8 to include encapsulated DNA sequences of larger size. Further, such sequences can be used in research to study and form genetic constructs, particularly, vectors, probes, components of gene editing systems, gene-therapy means, etc.

Encapsulation of genomic DNA into ZIF-8 distinguishes the present work among similar publications, in which nucleic acid fragments of fixed size were exposed to encapsulation [35–37, 55–57]. Confirmed feasibility of genomic DNA encapsulation through PCR genomic loci localized on different chromosomes makes this approach highly promising in the context of developing abiotic vectors for genetic material delivery. In particular, the capability of encapsulation of large-sized nucleic acids widens the potential of recombinant RNA technologies relieving the constraints on the useful load size typical for plasmid, cosmid, and viral vectors. However, relatively low efficiency of genomic DNA mineralization is not a barrier for further selection of targeted loci from the preserved genomic DNA, their cloning, modification, and other operations accompanying the development of recombinant sequences [32]. Moreover, the use of this non-toxic MOF as a carrier enables to “cushion” the problems with stability, toxicity, and immunogenicity, which is typical for other abiotic vectors. Thus, the study findings are of great importance for further development of research methodology in molecular genetics and gene engineering.

## Conclusion

The present study demonstrated the possibility of native DNA biomineralization into a model MOF ZIF-8. Optimal mole ratios of MOF components (Zn^2+^: 2-methylimidazole) for DNA biomineralization were found. The study of DNA@ZIF-8 composites by X-ray powder difractometry showed DNA to have no effect on crystalline structure of zinc 2-methylimidazole. Porosimetry enabled to demonstrate threefold decrease of the composite surface area compared to pure MOF, which is likely to be due to DNA inclusion. If mole ratios of the components were 1:40 and 1:60, the efficiency of genomic DNA encapsulation was found to be 8.58±1.50 and 7.94±1.25%, respectively, it providing DNA preservation in the amounts sufficient for further procedures. No significant differences between these mole ratios were revealed. The performed PCR analysis of arbitrary DNA loci after biomineralization and the composites dissolving demonstrated their preservation.

The findings indicate the approach prospectivity to develop vectors for delivering nucleic acids of arbitrary sizes and give grounds the feasibility of further studies in the field.
